# EEG Dataset for RSVP and P300 Speller Brain-Computer Interfaces

**DOI:** 10.1038/s41597-022-01509-w

**Published:** 2022-07-08

**Authors:** Kyungho Won, Moonyoung Kwon, Minkyu Ahn, Sung Chan Jun

**Affiliations:** 1 School of Electrical Engineering and Computer Science, Gwangju Institute of Science and Technology, 123 Cheomdangwagi-ro, Buk-gu, Gwangju, 61005 South Korea; 2 Bio and Medical Health Division, Korea Testing Laboratory, 87, Digital-ro 26-gil, Guro-gu, Seoul, 08389 South Korea; 3 School of Computer Science and Electrical Engineering, Handong Global University, 558 Handong-ro Buk-gu, Pohang, Gyeongbuk 37554 South Korea

**Keywords:** Electroencephalography - EEG, Attention

## Abstract

As attention to deep learning techniques has grown, many researchers have attempted to develop ready-to-go brain-computer interfaces (BCIs) that include automatic processing pipelines. However, to do so, a large and clear dataset is essential to increase the model’s reliability and performance. Accordingly, our electroencephalogram (EEG) dataset for rapid serial visual representation (RSVP) and P300 speller may contribute to increasing such BCI research. We validated our dataset with respect to features and accuracy. For the RSVP, the participants (N = 50) achieved about 92% mean target detection accuracy. At the feature level, we observed notable ERPs (at 315 ms in the RSVP; at 262 ms in the P300 speller) during target events compared to non-target events. Regarding P300 speller performance, the participants (N = 55) achieved about 92% mean accuracy. In addition, P300 speller performance over trial repetitions up to 15 was explored. The presented dataset could potentially improve P300 speller applications. Further, it may be used to evaluate feature extraction and classification algorithm effectively, such as for cross-subjects/cross-datasets, and even for the cross-paradigm BCI model.

## Abstract (summary)

**Table:** 

Measurement(s)	Electroencephalography
Technology Type(s)	electroencephalography (EEG)
Sample Characteristic - Organism	Homo sapiens

## Background & Summary

For many years, people have benefited from brain-computer interface (BCI) as a new non-muscular channel for communicating with the external world^1^. According to control signals, BCI can be divided into several types^2^; each type can provide a specific function, such as cursor control, virtual keyboard, and so on. Among the BCI applications, P300 speller is a popular BCI application Farwell and Donchin developed in 1988 that enters letters using brain activity^3^ called P300, which is one of the event-related potentials (ERPs) showing a positive deflection in EEG that appears approximately 300 ms in response to infrequent target stimuli^4–6^. As P300 has been reported to be highly stable^7^ and replicable^8^, the P300 speller yields stable performance and has helped people who require a communication tool that uses brain activity alone. In addition, classification accuracy has been improved by applying deep learning techniques to BCI, and many cross-subject models that use other participants’ training data have been proposed^9–11^. The BCI competition datasets have been used commonly to evaluate proposed model performance^12,13^; however, recently, datasets with a large number of participants have growing attention as benchmark datasets^14,15^. Such datasets are highly advantageous because of the amount of information available to train complex neural networks and transfer learning, and they are likely to have a broad performance distribution so that one could investigate whether a model works properly for a wide spectrum of participants. For example, Xu *et al*. examined cross-dataset variability and proposed a pre-alignment method across EEG dataset using eight BCI datasets containing various number of participants (average N = 27.13 ± 36.52, 4 to 109 participants)^16^. In this respect, we proposed another large EEG dataset that contains eyes-open/closed resting state, rapid serial visual presentation (RSVP), and visual P300-based BCI from 55 participants. Since our dataset contains rich information, such as eyes-open/closed resting states, questionnaire, and 3D electrode positions in addition to BCI data, it may be used to evaluate BCI performance with proposed classification methods and investigate the relation between default mode network and BCI performance. Further, it may be useful in developing data alignment methods across different datasets. We note that our dataset has been used already in our work on a P300 speller performance predictor^17^ and zero-training P300 speller^18^, but the dataset has not been published and has good potential implications for BCI research (see usage note section in the main text).

## Methods

### Participants

**Fig. 1:**
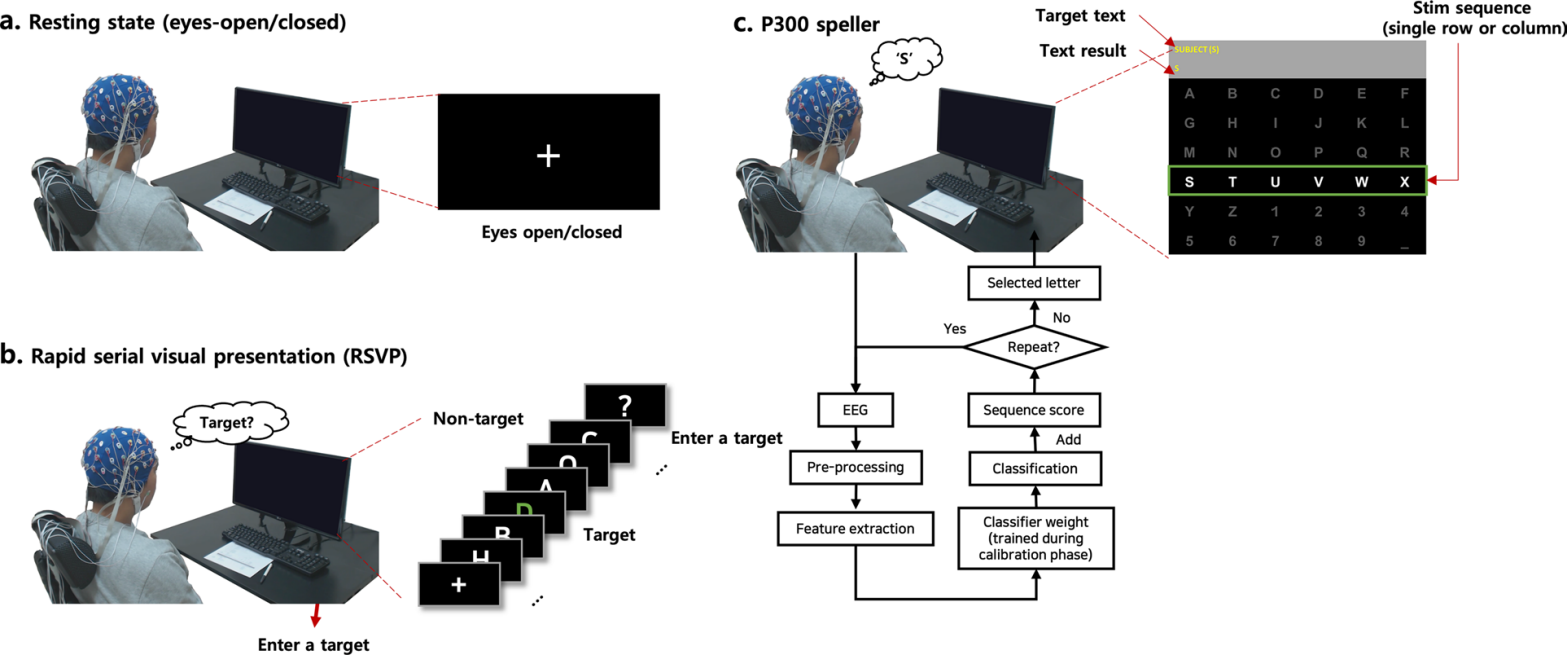
Experimental paradigm. (**a**) Resting state during eyes-open/closed, (**b**) rapid serial visual presentation (RSVP), and (**c**) P300 speller. The experimental procedure is described in Table 1.

We recruited a total of 55 participants (14 females, aged 22.91 ± 2.87) for a rapid serial visual presentation (RSVP) and P300 speller tasks. The Institutional Review Board of Gwangju Institute of Science and Technology (20171106-HR-31–01–02) approved the experiment, and all the participants were informed about the experiment and signed a written consent form beforehand. All participants performed the same experimental tasks, including eyes open/closed resting state, rapid serial visual presentation (RSVP), and a P300 speller task (Fig. 1). Each participant was seated in a comfortable chair approximately 1 m away from a 27-inch monitor screen in a room. The operator monitored the participant outside the room using a front webcam and delivered instructions by voice. The entire experimental procedure is summarized in Table 1.

**Table 1:** Detailed procedure in the experimental paradigm.

Number	Task	Duration (min)
1	Sign a consent form and complete a questionnaire	5
2	Equip EEG and test record	20
3	Digitize 3D electrode position	20
4	Resting state (eyes-open)	2
5	Resting state (eyes-closed)	2
6	Rapid serial visual presentation	6
7	Resting state (eyes-open)	2
8	Resting state (eyes-closed)	2
9	P300 speller - calibration phase RUN 1	4
10	P300 speller - calibration phase RUN 2	4
11	Complete a questionnaire	2
12	P300 speller - test phase RUN 1	6
13	Complete a questionnaire	2
14	P300 speller - test phase RUN 2	6
15	Complete a questionnaire	2
16	P300 speller - test phase RUN 3	6
17	Complete a questionnaire	2
18	P300 speller - test phase RUN 4	6
19	Complete a questionnaire	2
20	Resting state (eyes-open)	2
21	Resting state (eyes-closed)	2
22	Disengage and clean EEG	20
SUM		125

### Data acquisition

**Fig. 2:**
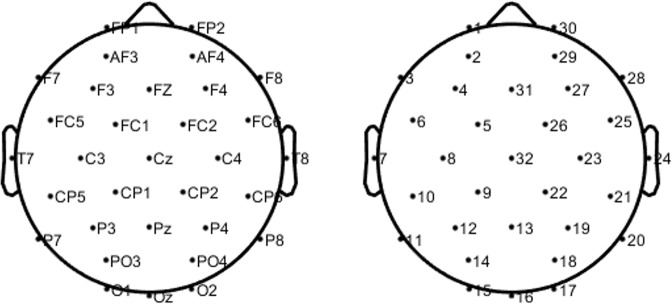
Electrode channel configuration. Electrode labels (left) and their corresponding numbers (right).

EEG data were collected at a 512 Hz sampling rate using 32 Ag/AgCl active electrodes according to the international 10–20 system (see Fig. 2 for electrode names and the corresponding channel indices). The EEG device used in this experiment was the Biosemi ActiveTwo system. EEG acquisition and stimulation tasks (resting state, RSVP, P300 speller tasks) were conducted with BCI2000 software^19^. Before the first resting state was recorded, we collected EEG channel locations (electrode 3D coordinates) using a 3D coordinate digitizer (Polhemus Fastrack) for each participant. The measured channel locations were determined as the average of two measurements of the digitizer to avoid handshaking.

### Resting state

During the resting state, the participants were instructed to minimize their body movement, stay relaxed for 2 minutes, and keep their eyes open and eyes closed, respectively. For the eyes-open state, the participants were instructed to stare at a fixation cross on the screen; for the eyes-closed state, the participants were instructed to close their eyes until the end of the recording session. The operator spoke to the participants at the end of the recording session. The resting state was collected three times— before and after the RSVP task (before the first P300 speller calibration run), and after the last run of the P300 speller.

### Rapid serial visual presentation (RSVP)

**Fig. 3:**
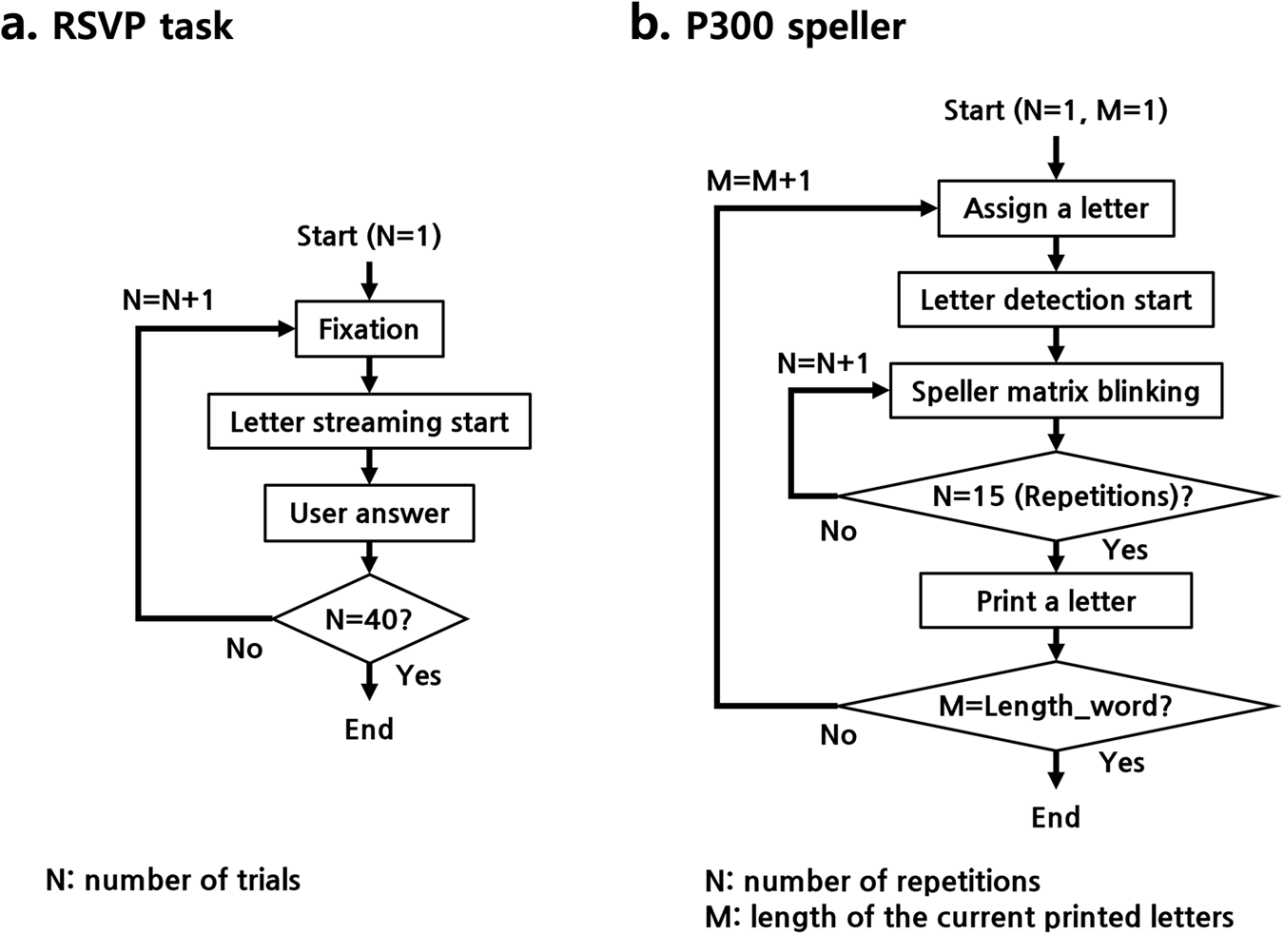
Flowcharts for the RSVP and P300 speller tasks. (**a**) represents RSVP task flow, and (**b**) represents P300 speller task flow.

A rapid serial visual presentation (RSVP) task is one in which a participant detects a single target letter or image in a rapidly refreshing letter or image stream at the same location^20,21^. The RSVP task is known to elicit ERPs when a participant focuses selectively on a target and ignores non-targets^20^. Figure 3a represents the RSVP task procedure from start to end. Specifically, as illustrated in Fig. 1b, participants were instructed to press the keyboard to recall the target character within 5 seconds after each character stream consisting of one target character (green-colored) and 20 non-target characters (white-colored) was displayed with a 10 Hz refresh rate. The participants performed 40 RSVP trials, such that there were 40 target events and 800 non-target events for each participant. The detection accuracy of target letters (the number of target characters identified correctly among 40 RSVP trials) was defined as T1%. During the task, the participants received no feedback on whether they identified the target characters correctly.

### P300 speller

During the P300 speller task, the participants were instructed to spell the target text through Farwell and Donchin’s 6 × 6 matrix-based speller^3^ that consists of alphabet letters (A–Z), digits (1–9), and space (“_”), as shown in Fig. 1c. In general, the P300 speller does not print letters using a single trial; instead, it uses an ERP called P300 elicited by the target sequence (single row and column) among blinking sequences. Thus, the speller matrix consists of 12 sequences, 6-row and 6-column sequences (hereinafter we refer to a single row or column sequence as a stim sequence). At the beginning of a letter block, the stim sequence blinks white and dark gray in random order. For each blink, the trained classifier determines whether the blinked stim sequence is target or non-target, then stores the classifier output (target or non-target; corresponding sequence column or row index). To enhance the signal-to-noise ratio (SNR), the operators could set the number of the stim sequences’ repetitions. At the end of the letter block, the speller application determines the final target row and column with the highest scores among the 6 row and column sequences. Finally, a single letter is printed and begins the next block. Figure 3b represents the P300 speller task procedure for a single word block, and step by step explanation follows.

In this experiment, BCI2000 software^19^ was used for P300 speller application and EEG data acquisition during the task. As depicted in Fig. 1c, the P300 speller consists of the target text region, text results (classified letters) region, and speller matrix region. Each stim sequence was flashed for 125 ms and turned off for 62.5 ms until the next sequence was flashed. In this experiment, each stim sequence was blinked 15 times to print a single character. Therefore, each target character blinked a total of 30 times and non-target characters blinked a total of 150 times. Participants stared at the speller matrix, attended to the target letter blinks (target stim sequence), and ignored other blinks (non-target stim sequences). During the two calibration runs, the participants were instructed to print two words (“BRAIN” and “POWER”), but visual feedback was not provided. After the calibration runs, classifier weights were trained using stepwise linear discriminant analysis (SWLDA) with BCI2000 software^22,23^. To train the classifier, EEG 800 ms from the stimulus onset were extracted and down sampled to 20 Hz. The best 60 features among the features were chosen to detect the P300. In the test runs, the participants were instructed to print four words (“SUBJECT”, “NEURONS”, “IMAGINE”, and “QUALITY”). Visual feedback was provided on the top of the P300 speller, as depicted in Fig. 1c.

We note that this dataset includes 17 unique letters (from four test words) among 36 letters (from 6 × 6 speller matrix). In P300 speller, the trained classifier determines only whether the upcoming sequence is target or non-target regardless of target letter location. In other words, in Fig. 1c, letter “A” (1st row, 1st column) and letter “_” (6th row, 6th column) has the same binary classification problem. Therefore, P300 speller dataset does not need to spell all the letters and other research studies instructed to spell some part of letters from their own sentences^15^. On the other hand, there is another BCI speller type controlled by steady-state visual evoked potential (SSVEP)^24^, which is an EEG component elicited by a visual stimulus that is modulated at a fixed frequency. Because each letter is coded with distinct frequency in SSVEP speller^25,26^, letter location for the SSVEP speller may be a factor associated with accuracy and thus all letters in the speller should be tested because each letter has a unique frequency and phase stimulus.

### Questionnaire

In addition to acquiring EEG data, the participants’ physical/mental states were collected using the questionnaire shown in Table 2. We note that the questionnaire was written in Korean first and then translated into English. The participants completed the questionnaire before the experiment (number 1 to 21), after the P300 calibration phase (number 22 to 34) after each P300 test run (number 35 to 47, 48 to 60, 61 to 73, and 74 to 84, respectively), and at the end of the experiment (number 85 to 88). All answers collected (numerical values or characters) to the questions were stored in a single file (*.xlsx), and unanswered questions were marked as the numeric value of 0.

**Table 2:** Questionnaire.

Number	Question							
**A. Individual information**								
1	Handedness (Right = R, Left = L)							
2	Diseases (Yes = Y, No = N)							
3	Age (number)							
4	Gender (Male = M, Female = F)							
5	Normal vision or corrected (lens, glasses) vision (Corrected = Y, normal = N)							
6	Notes (BCI or biofeedback experience) - Yes = Y, No = N							
**B. Before experiment**								
7	1. Are you curious about today’s experiment?							
8	2. Are you willing to try your strategy for effective experimental training?							
9	3. Do you look forward to achieving a high score (P300 speller performance)?							
10	4. Are you proud of yourself for achieving a high score?							
11	5. Are you interested in the fact that people can communicate using brain waves?							
12	6. How long did you sleep last night? (hours)							
13	7. Did you drink coffee within 24 hours?							
14	8. Did you drink liquor within 24 hours?							
15	9. Did you smoke within 24 hours?							
	10. How do you feel?							
16	10.1 Anxiety	Anxious	1	2	3	4	5	Relaxed
17	10.2 Boredom	Bored	1	2	3	4	5	Excited
18	10.3 Physical state	Very bad and tired	1	2	3	4	5	Very good
19	10.4 Mental state	Very bad and tired	1	2	3	4	5	Very good
20	10.5 Eye state	Dry and stiff	1	2	3	4	5	Very good
21	11. How do you predict your overall BCI performance? (in %)							
**During P300 speller task (C-D)**								
**C. After Calibration run (after P300 speller calibration run02)**								
22	1. Could you continue the next run? (Yes = Y, No = N)							
	2. How do you feel?							
23	2.1 Anxiety	Anxious	1	2	3	4	5	Relaxed
24	2.2 Boredom	Bored	1	2	3	4	5	Excited
25	2.3 Concentration	Very bad	1	2	3	4	5	Very good
26	2.4 Physical state	Very bad and tired	1	2	3	4	5	Very good
27	2.5 Mental state	Very bad and tired	1	2	3	4	5	Very good
28	2.6 Eye state	Dry and stiff	1	2	3	4	5	Very good
29	3. Were you sleepy during the task?	Not sleepy	1	2	3	4	5	Very sleepy
30	4. Was it too fast?	Totally disagree	1	2	3	4	5	Totally agree
31	5. Was it too difficult?	Totally disagree	1	2	3	4	5	Totally agree
32	6. Was it easy to concentrate on the task?	Very difficult	1	2	3	4	5	Very easy
33	7. How do you predict your performance for the last run? (in %)							
34	8. How do you predict your performance for the next run? (in %)							
**D. After P300 speller test run01 to run04 (4 repetitions)**								
run01 (35~47), run02 (48~60), run03 (61~73), run04 (74~84) run 04 – no 1 and 7	1. Could you continue the next run? (Yes = Y, No = N)							
	2. How do you feel?							
	2.1 Anxiety	Anxious	1	2	3	4	5	Relaxed
	2.2 Boredom	Bored	1	2	3	4	5	Excited
	2.3 Concentration	Very bad	1	2	3	4	5	Very good
	2.4 Physical state	Very bad and tired	1	2	3	4	5	Very good
	2.5 Mental state	Very bad and tired	1	2	3	4	5	Very good
	2.6 eye state	Dry and stiff	1	2	3	4	5	Very good
	3. Were you sleepy during the task?	Not sleepy	1	2	3	4	5	Very sleepy
	4. Was it too fast?	Totally disagree	1	2	3	4	5	Totally agree
	5. Was it too difficult?	Totally disagree	1	2	3	4	5	Totally agree
	6. Was it easy to concentrate on the task?	Very difficult	1	2	3	4	5	Very easy
	7. How do you predict your performance for the next run? (in %)							
**E. After the experiment**								
	1. How was today’s experiment?							
85	1.1 Duration	Too short	1	2	3	4	5	Too long
86	1.2 BCI application evaluation	Very bad	1	2	3	4	5	Very good
87	1.3 Experimental environment	Uncomfortable	1	2	3	4	5	Comfortable
88	1.4 Task difficulty	Difficult	1	2	3	4	5	Easy

### Preprocessing and feature extraction

In preprocessing, we applied the minimum processing conventionally necessary, in that additional preprocessing remains at the user’s discretion. This experiment involves real-time P300 speller runs; in practice, complex signal processing is applied rarely because the online procedure requires considerable time, and a decision is likely to be made regardless of whether the current epoch is good or bad. Thus, most investigators apply their preferred preprocessing pipeline in the offline analysis, such as rejecting bad epochs. In our case, during preprocessing, we first re-referenced the EEG data with common average reference (CAR) that uses all electrode channels as a reference because the EEG device (Biosemi ActiveTwo system) used for data acquisition does not provide hardware-level referencing. Here, we validated the EEG data collected during the RSVP and P300 speller tasks. For the resting state, no data validation was considered since resting state EEG records default brain activity from the participants when they did nothing. Therefore, users can decide how to analyze this EEG upon their analysis purpose.

### RSVP

RSVP includes the participant’s keyboard response (defined as T1%) and ERPs for 40 trials. To calculate ERPs, EEG data were band-pass filtered with the bandwidth of [1 10] Hz as one of the conventional bandwidths for the P300 detection^27^ to remove noise and preserve P300 information. Further, high pass filtering (≥1 Hz) was applied to increase signal-to-noise ratio (SNR) by removing noise caused by non-brain activities, such as motion artifacts, and low pass filtering (≤10 Hz) was applied to remove artefacts induced by 10 Hz refresh rate. However, we note that low pass filtering with higher cut-off frequency yielded no difference. Then, the filtered data were extracted with [−200 1000] ms relative to the stimulus onset, and baseline correction was performed with [−200 0] ms before onset. Specifically, 6 adjacent non-target epochs (after or before target) were removed for each stream as the target and non-target epochs may overlap because of the rapid refresh rate. As a result, it yielded extracted epochs of every participant with dimensions of [32 × 615 × 40] for targets and [32 × 615 × 560] for non-targets, where ‘32’ represents electrode channels, ‘615’ represents samples (512 Hz × 1200 ms), and ‘40’ and ‘560’ represent the number of targets and non-targets in the RSVP, respectively. Before the trials were averaged to display ERPs, certain epochs with an amplitude greater than ±100 μV except for the frontal electrodes close to the eyes (FP1, FP2, AF1, and AF3) were removed.

### P300 speller

ERPs during P300 speller sessions were examined just as those in RSVP. Next, we evaluated the P300 speller performance with respect to classification accuracy as the number of stim sequence repetitions varied. The P300 speller data included 2 calibration runs (2 of 5-letter words) and 4 test runs (4 of 7-letter words). For validation purposes, to extract features, we used EEG from the test runs only because of its simplicity, as there was no visual feedback during the calibration runs. Because the P300 speller and RSVP elicit the same EEG characteristics, we calculated ERPs of the P300 speller in the same manner as for RSVP. However, epoch removal was not considered for the P300 speller data. We believe that additional processing in an online setting (BCI test runs) may not be necessary and may be done when needed at the user’s discretion. As a result, it yielded extracted epochs with a dimension of [32 × 615 × 840] for targets and [32 × 615 × 4200] for non-targets for every participant, where ‘32’ represents electrode channels, ‘615’ represents samples (512 Hz × 1200 ms), and ‘840’ and ‘4200’ represent the number of targets and non-targets in the P300 speller, respectively.

In addition to P300 speller features, performance was assessed according to offline letter detection accuracy. As stated in the experimental design section, the P300 speller outputs a letter by collecting several stim sequences and classifying them as targets or non-targets. Eventually, the row and column indices in a 6 × 6 matrix that yield the highest scores (i.e., those classified as a target most frequently in given row/column stims, respectively) were used to print a target letter. In addition to online P300 speller performance, we calculated offline performance over various repetitions (1 to 15). First, a classification model was trained using the EEG collected during calibration runs. Training data were bandpass-filtered at [0.5 10] Hz, as the same as the RSVP EEG preprocessing – high pass filtering to increase signal-to-noise ratio (SNR) by removing noise caused by non-brain activities, such as motion artifacts and low pass filtering to remove SSVEP effects that may be induced by the constant blinking speller matrix. Compared to the RSVP task, P300 speller task was more stable in EEG because the RSVP task did require keyboard press to answer, whereas P300 speller did not require any body movement. As a result, in P300 speller, noise (inspected visually) was observed to be minimized with 0.5 Hz cut-off frequency for high pass filtering, in place of 1 Hz. We note that low pass filtering with higher cut-off frequency yielded no difference. Afterwards, epochs [0 600] ms from the stimulus onset were extracted and baseline corrected from 200 ms prior to the onset. To increase the signal-to-noise ratio (SNR), epochs were down-sampled from 512 Hz to approximately 20 Hz by averaging 24-time points without overlap, which resulted thereby in 32 (channels) × 12 (down-sampled time points). Finally, the epochs extracted to train the model had a dimension of [1800 × 384] for each participant. Here, 1800 indicates the number of targets (300) and nontargets (1500), and 384 indicates the concatenated features of 32 channels ×12-time samples. Then, the epochs extracted were used to train the stepwise linear discriminant analysis (SWLDA) model. SWLDA includes the step of adding and removing features depending upon their contribution to the classified labels^22,23^, so it can reduce the feature space from the concatenated feature vector to the reduced feature vector. Among 384 features, the best 60 features were used to train classifier weights. After training the classification model, the EEG data collected during the test runs were used to evaluate the P300 speller performance. During the test runs, 4 words of 7 letters, i.e., a total of 28 letters to spell, were presented. The test data were processed in the same manner as the training data, so the epochs extracted for the test had a dimension of [5040 × 384]. Here, 5040 indicates the number of targets (840) and nontargets (4200), and 384 indicates the concatenated features. Then, every letter was printed using every 180 epochs (30 targets, 150 nontargets), and letter detection accuracy was calculated as the number of letters printed correctly. In addition to using all of the epochs, the letter detection accuracy was estimated for a smaller number of repetitions (from 15 to 1), which yielded 180, 168, …, 12 epochs per letter because the 6 × 6 matrix speller has 12 stim sequences (6 rows and 6 columns).

## Data Records

The EEG data and questionnaire data are downloadable from the open access repository – figshare^28^. The MATLAB-compatible resource consists of 55 EEG-data files (a total of approximately 13.74 GB). Each file is named as participant codes (s01 to s55). The data have a type of MATLAB structural cell array and are formatted to (*.mat, -v7.3) extension that can be loaded using MATLAB and Python (mat73 module) for each participant. The detailed data structure is described as below:


EEG = a struct with fields:• RSVP: data structure that contains details of RSVP task•train: cell array in which element represents the data structure for each calibration run• test: cell array in which element represents the data structure for each test run• rest: cell array in which element represents the data structure for resting sates (fields: open [ch × time], close [ch × time])• senloc: data structure of electrode positions (fields: electrodes_pos [ch × (x, y, z)], labels [1 × ch])EEG.RSVP =a struct with fields:• accuracy_t1: accuracy pressed correctly for RSVP targets• nbTrials: the number of RSVP trials• nbTrials_target: the number of target trials• nbTrials_nontarget: the number of non-target trials• data: EEG ([ch × time])• srate: sampling rate• markers_target: event markers• chanlocs: channel location including labels• keyboard_response: keyboard press response• target: pre-defined RSVP targetsEEG.train/EEG.test = % For EEG.train and EEG.test; their runs are separated in the form of a cell array.a struct with fields:• nbTrials: the number of trials• nbTrials_target: the number of target trials• nbTrials_nontarget: the number of non-target trials• data: EEG ([ch x time])• srate: sampling rate• markers_seq: event markers (letters to spell)• markers_target: event markers (target and non-target)• text_to_spell: predefined text to spell• text_result: user result• online_acc: letter detection accuracy (0 for train EEG)• chanlocs: channl location including labels


In addition to this data structure format, we provided the dataset with EEG-brain imaging data structure (BIDS)^29,30^ on the same repository (a total of approximately 9.15GB)^28^ which has rich information for the dataset so that most BCI investigators could organize and share EEG data easily between laboratories.

## Technical Validation

### RSVP features and performance

**Fig. 4:**
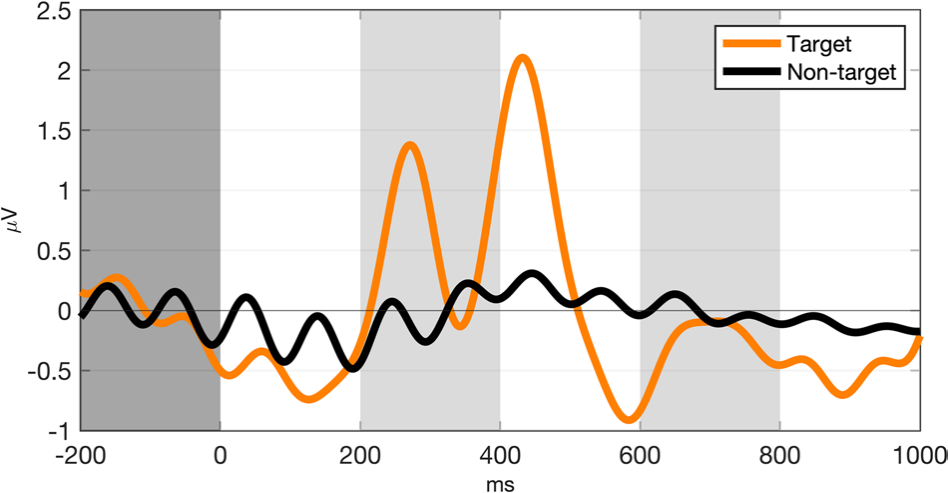
RSVP ERP. The grand-averaged ERP response at the midline (Fz, Cz, and Pz) during RSVP.

**Fig. 5:**
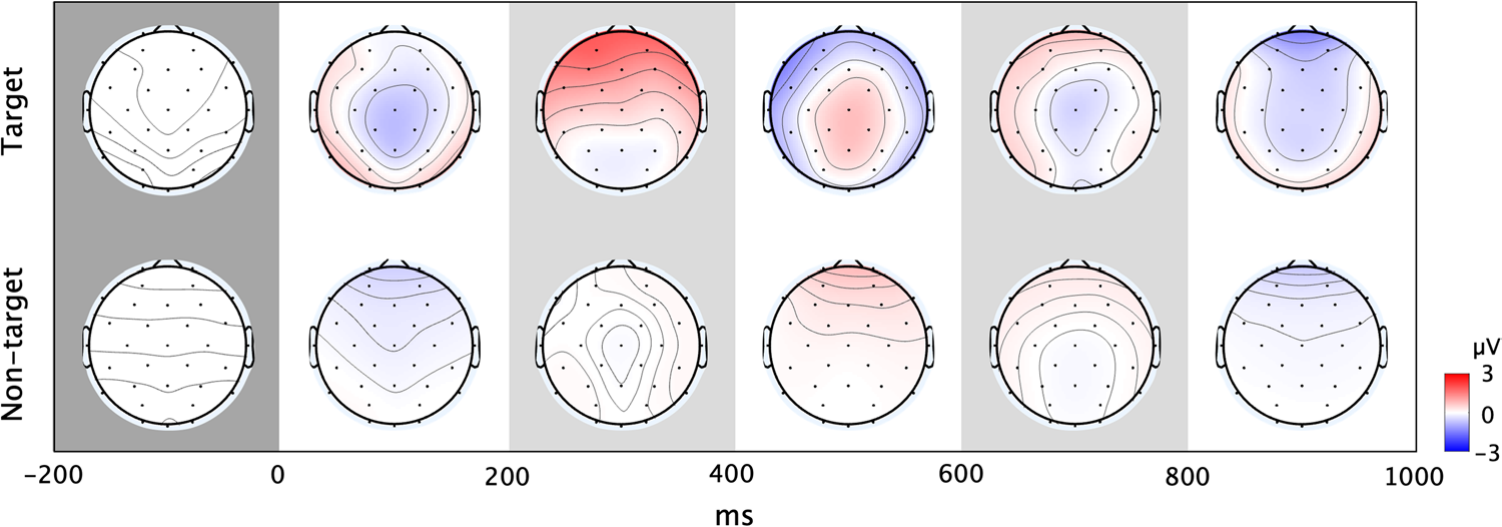
RSVP ERP topography. The grand-averaged ERP scalp topography over time during RSVP.

Five participants’ (s43 to s47) RSVP responses (T1%) were not recorded because the keyboard malfunctioned; however, their EEG data were recorded. With respect to RSVP T1%, the remaining 50 participants achieved 91.85 ± 5.6% (77.5–100%), while the RSVP EEG analysis was performed with data from all participants. Within a single participant’s epochs, up to 10% and 10.54% were removed from target and non-target events, respectively, after trials that had an absolute amplitude greater than 100μV were rejected. Individually, P300 amplitude (defined as the peak amplitude within an epoch) was 3.7782 ± 2.1450 μV and P300 latency (defined as peak latency) was 315.12 ± 84.93 ms. Figure 4 represents the grand-averaged ERP waveform during RSVP, and Fig. 5 represents ERP scalp topography plots over time during target and non-target events. As shown in Fig. 4, we observed that clear peaks appeared at 200–600 ms around the midline (Fz, Cz, and Pz) during target events, while the waveform during non-target events was not notable. With respect to scalp topography, ERPs appeared at the fronto-central to parietal areas during target events, while there were only relatively small changes in amplitudes during non-target events, which is consistent with reported work^4,6^.

### P300 speller features and performance

**Fig. 6:**
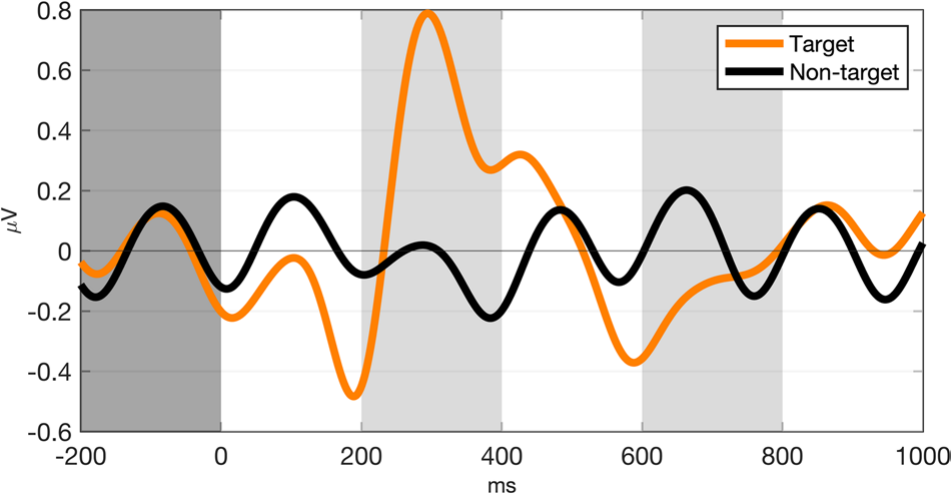
P300 speller ERP. The grand-averaged ERP response at the midline (Fz, Cz, and Pz) during P300 speller test sessions.

**Fig. 7:**
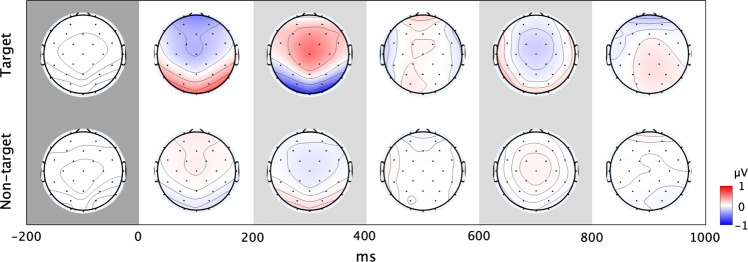
P300 speller ERP topography. The grand-averaged ERP scalp topography over time during P300 speller test sessions.

The P300 speller EEG analysis was performed on all participants. Individually, the P300 peak amplitude was 0.9155 ± 0.3668 μV and the peak latency was 261.51 ± 43.02 ms. Compared to RSVP, the P300 speller ERPs had a lower amplitude and shorter latency. It is unsurprising that the trial average yielded weaker average amplitudes because the P300 speller has many more target epochs than those of RSVP. Figure 6 represents the grand-averaged ERP waveform during P300 speller test runs, while Fig. 7 shows ERP scalp topography plots over time during target and non-target events. As illustrated in Fig. 6, we observed a clear positive peak at 200–400 ms around the midline (Fz, Cz, and Pz) during target events, while the waveform during non-target events was not evident. With respect to scalp topography, ERPs were evoked at the fronto-central to parietal areas during target events, while there were only small changes in amplitudes during non-target events, as observed in RSVP. Overall, we observed similar EEG characteristics during target events in both RSVP and P300 speller.

**Fig. 8:**
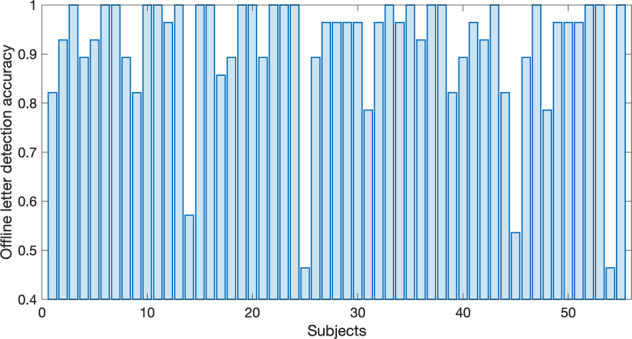
P300 speller letter detection accuracy. Distribution of P300 speller offline letter detection accuracy for 55 participants.

**Fig. 9:**
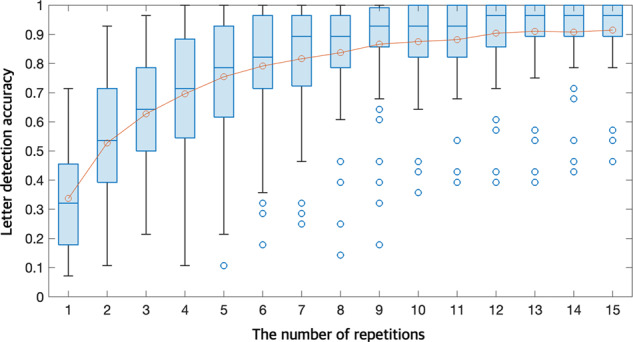
P300 speller letter detection accuracy according to the number of repetitions. Subject-averaged letter detection accuracy. Each boxplot indicates letter detection accuracy from 55 participants according to the number of repetitions. Orange circles denote the average letter detection accuracy and blue circles indicate outliers in box plots.

All participants’ P300 speller performance was evaluated for 4 words (“SUBJECT”, “NEURONS”, “IMAGINE”, and “QUALITY”). Figure 8 represents their letter detection accuracy, which was 91.49 ± 13.12% (46.43–100%). We found that 49 of 55 participants achieved performance higher than 80%, while four participants only achieved a performance lower than 60%. It is because 32 whole-head electrode channels and the number of epochs used in this experiment were sufficient to achieve the advantages of ensemble classification. Thus, we investigated the letter detection accuracy in the number of epochs per letter (the number of repetitions) from 1 to 15. As shown in Fig. 9, letter detection accuracies were estimated from 33.70 ± 16.65% to 91.49 ± 13.12%, as the number of repetitions varied from 1 to 15. From this investigation, we may presume that if only 85% accuracy is necessary to operate the P300 speller, 9 rather than 15 repetitions are sufficient. Further, the improvement in letter detection accuracy was marginal after 12 repetitions, so additional repetitions appear to be unnecessary. As expected, a reduction in the number of repetitions increased P300 speller speed and decreases accuracy; thus, this investigation may provide a reasonable tradeoff between repetition and accuracy.

## Usage Notes

In this note, we proposed our benchmark dataset for the P300 speller collected from 55 participants, including RSVP and resting state EEG, a questionnaire, and 3D electrode positions. To show the dataset’s reliability, minimal preprocessing was performed to extract ERPs from RSVP and the P300 speller, after which the P300 speller performance was evaluated. The results showed clear ERPs and a reasonable distribution in P300 speller performance. Thus, we believe that any investigation may be conducted without difficulty by applying any processing pipeline and classification algorithm. With respect to pre-processing, high order statistics, such as independent component analysis (ICA) to remove artifact components^31^, and automatic artifact removal^32^ may be applied accordingly. In our previous work^17,18^, we used the dataset to investigate the relation between the P300 speller performance and EEG characteristics during similar cognitive tasks (RSVP)^17^, and also used a large number of participants to propose a cross-subject classifier using a convolutional neural network (CNN)^18^.

Further, the dataset has the potential to build cross-subject^10,33,34^, cross-dataset^16^, and cross-paradigm^35^ classifiers, and it may be useful when investigating the relation between the default mode network and attention level or P300 speller performance^36^ to optimize the P300 speller’s speed and accuracy. In addition, well-designed questionnaires and 3D electrode positions are quite useful for more in-depth investigation of neurophysiological and psychological aspects. Recently, with the growing necessity to have open datasets and trustworthy algorithms, the open-source framework, including the collection of open EEG datasets, has been proposed^37^, and relevant research studies have used the dataset to validate their algorithms^16^. With respect to building classifiers, with the development of deep learning and advanced signal processing, many zero-training BCI techniques have been proposed. A large EEG dataset can definitely contribute to comparing the proposed zero-training BCI techniques more reliably, compared to datasets that have a small number of participants because EEG has high inter-subject variability.

### Ethical approval

The Institutional Review Board at Gwangju Institute of Science and Technology approved this experiment(20171106-HR-31–01–02).

## Data Availability

Project name: EEG dataset for RSVP and P300 Speller Brain-Computer Interfaces. Project home page: https://github.com/KyunghoWon-GIST/EEG-dataset-for-RSVP-P300-speller. Operating system(s): Windows, MAC. Programming language: MATLAB, Python. Other requirements: MATLAB r2020a or higher, Python 3.6 or higher. License: MIT License. We note that the results of the article were produced using MATLAB. We provide MATLAB and Python scripts, and users can use Python to extract features and evaluate P300 speller performance as well, but the result may differ slightly from MATLAB.
